# Actions of N-acetylcysteine, daptomycin, vancomycin, and linezolid on methicillin-resistant *Staphylococcus aureus* biofilms in the ventriculoperitoneal shunt infections: an experimental study

**DOI:** 10.1186/s41016-022-00284-2

**Published:** 2022-07-05

**Authors:** Tuba Kuruoglu, Gamze Altun, Enis Kuruoglu, Derya Bayırlı Turan, Mehmet Emin Önger

**Affiliations:** 1 Department of Infectious Diseases and Clinical Microbiology, Medical Faculty, Ondokuz Mayıs University, Samsun, Turkey; 2 Department of Histology and Embryology, Medical Faculty, Ondokuz Mayıs University, 55139 Samsun, Turkey; 3 Department of Neurosurgery, Medical Faculty, Ondokuz Mayıs University, Samsun, Turkey; 4 Department of Infectious Diseases and Clinical Microbiology, School of Medicine, Yeni Yuzyil University, Istanbul, Turkey; 5 Department of Neuroscience, Health Science Institute, Ondokuz Mayıs University, Samsun, Turkey

**Keywords:** Methicillin-resistant *Staphylococcus aureus*, Biofilm, Antibiotic-impregnated catheters, Scanning electron microscope

## Abstract

**Background:**

Shunt systems are used to provide cerebrospinal fluid drainage in the treatment of hydrocephalus. Recently, antibiotic-impregnated shunt systems are used to prevent colonization in the ventriculoperitoneal catheters. Methicillin-resistant *Staphylococcus aureus* (MRSA) is the most common causative microorganism of shunt infections. The aim of the study is to investigate effects of several substances on MRSA biofilms in the ventriculoperitoneal catheters.

**Methods:**

The present study consists of mainly eight groups (each has two subgroups as antibiotic-impregnated and nonantibiotic-impregnated catheters). In addition, each group contains six molds using MRSA strains. In this study, daptomycin (DAPT) (2 mg/ml), vancomycin (VAN) (10 mg/ml), linezolid (LIN) (2 mg/ml), N-acetylcysteine (NAC) (6 mg/ml), and various combinations of these substances were used to evaluate the treatment against MRSA using scanning electron microscope (SEM) images and microbiological enumeration.

**Results:**

The colony count in the antibiotic-impregnated samples significantly decreased compared to nonantibiotic-impregnated samples in the MRSA, MRSA + DAPT, and MRSA + LIN groups (*p* < 0.01), respectively. Conversely, the colony count in antibiotic-impregnated samples significantly increased compared to nonantibiotic-impregnated samples in NAC + DAPT and NAC + VAN groups (*p* < 0.01), respectively.

**Conclusions:**

The results showed that the use of antibiotic-impregnated catheters has a significant impact on the prevention of infection whereas the combination of NAC and DAPT showed better antibiofilm and antibacterial effects than other combinations on the prevention and treatment of nonantibiotic-impregnated catheter infections.

## Background

For several years, shunt systems providing cerebrospinal fluid drainage are widely used in the treatment of hydrocephalus. The most common neurosurgical disease in children seen in 3 to 5 infants of 1000 live births is hydrocephalus. Among complications related to ventriculoperitoneal shunt in hydrocephalus treatment, shunt infections and obstructions are seen at high rates [1]. About 10% of shunt infections show a risk for general infection mainly by the bacterial strains *Staphylococcus aureus* (*S. aureus*) and Enterococci [2]. Generally, most shunt infections occur during the surgical procedure and commonly within the first few months following surgery [3]. The duration of systemic antibiotic treatment varies depending on the cerebrospinal fluid sterilization rate and the type of isolated microorganism. However, there is no adequate controlled randomized prospective study. Treatment usually continues for 7–10 days for the coagulase-negative staphylococcal strains [4].

Determining the etiology and antimicrobial resistance profile of these infections caused by invasive pathogens is very important in identifying and explaining prevention strategies and determining the clinical treatment. The most common pathogens causing the infection are coagulase-negative Staphylococci (50 to 90%) and *S. aureus* (13 to 27%). Other organisms involved are aerobic gram-negative bacilli, Streptococci, and Propionibacterium [5]. The bacteriostatic activity of linezolid (LIN) against MRSA has also been reported [6]. In addition, in 6 of 12 patients with bacteremia caused by *Staphylococcus aureus*, a decreased susceptibility to vancomycin (VAN) was reported using LIN alone or in combination with fusidic acid and rifampin [7]. In this context, Brown et al. suggested that VAN and LIN could be used to treat the shunt infections caused by *Staphylococcus* spp. [5]. When LIN and VAN were compared, intravenous administration with LIN could achieve therapeutic cerebrospinal fluid (CSF) levels, while intraventricular administration of VAN was required to achieve the therapeutic CSF levels. This administration may be considered an advantage of LIN compared with VAN.

The advantage of LIN over VAN is that therapeutic CSF levels can be achieved by intravenous administration. Conversely, VAN must be administered intraventricular and may require a contra-lateral reservoir insertion to ensure success [8]. However, according to Lee et al. (2012), VAN was more effective than cefazolin in treating shunt infections. Besides, VAN is considered a prophylactic agent in shunt surgery [3]. However, VAN is increasingly questioned about the treatment of choice for MRSA bacteremia due to several factors, with the isolates’ reduced susceptibility being one reason.

Alternative therapies for bacteremia by MRSA are limited. The other treatment considered for *S. aureus* bacteremia is a bactericidal cyclic lipopeptide, daptomycin (DAPT) [9]. DAPT showed a bactericidal effect against MRSA bacteremia caused by endocarditis. Despite elevated minimum inhibitory concentration (MIC), the bactericidal concentration remained constant [10–12]. However, several studies suggested that VAN is considered the perioperative prophylactic agent in the MRSA shunt infections caused during re-inserting or inserting the shunt following the hydrocephalus. In addition to antibiotic treatment used following shunt insertion, it is also essential to use antimicrobial active shunt materials [3, 13].

The biofilm layer formed by staphylococci is one of the most important reasons that complicates the effectiveness of antibiotics in treatment. N-acetylcysteine (NAC) is a molecule with antioxidant and mucolytic properties that inhibits growth, adhesion, and biofilm formation of gram-positive bacteria [14] The usage of NAC continues in various studies to prevent biofilm-induced antibiotic resistance and to increase the effectiveness of antibiotics used in the treatment [15].

Due to its high morbidity and mortality, understanding the pathophysiology of the causative microorganisms of shunt infection is important to prevent and manage bacteremia [16, 17]. Thus, our study aimed to investigate the infection rate of ventriculoperitoneal shunt infections caused by MRSA and discuss the different pharmaco-therapeutic treatments.

## Methods

In this study, antimicrobial and antibiofilm effects of NAC alone and in combination with DAPT, VAN, and LIN, respectively, on MRSA biofilm in rifampicin-clindamycin impregnated and nonimpregnated ventriculoperitoneal shunts were investigated.

### Biofilm formation

Two different kinds (antibiotic-impregnated as rifampicin (0.54%)–clindamycin (15%) and nonantibiotic-impregnated) of commercial ventriculoperitoneal shunts (Bactiseal®, Codman) were used in this study. All shunts were cut into small pieces as 0.5 mm in length and were infected with *Staphylococcus aureus* strain ATCC 43,300. The study consisted mainly of eight groups (each with two subgroups as antibiotic-impregnated and nonantibiotic-impregnated catheters used) (Table 1). All the pieces were placed in human plasma and incubated at 37 °C with shaking for 24 h. After the pieces were washed with 0.9% NaCl, they were placed in a suspension of MRSA (ATCC 43,300) with a turbidity equivalent to the 5 McFarland standard (~ 10^8^ cfu/ml) with tryptic soy broth (TSB; Merck, Darmstadt, Germany) supplemented with 0.5% glucose for an additional 24 h at 37 °C to induce biofilm formation [18].

**Table 1:** Group design where ( +) indicates the antibiotic-impregnated catheters and ( −) indicates the nonantibiotic-impregnated catheters

Study design		
**Group I**	**–**	Only MRSA infected
	** + **	
**Group II**	**–**	MRSA + NAC
	** + **	
**Group III**	**–**	MRSA + DAPT
	** + **	
**Group IV**	**–**	MRSA + VAN
	** + **	
**Group V**	**–**	MRSA + LIN
	** + **	
**Group VI**	**–**	MRSA + NAC + DAPT
	** + **	
**Group VII**	**–**	MRSA + NAC + VAN
	** + **	
**Group VIII**	**–**	MRSA + NAC + LIN
	** + **	

### Antibiotic treatment

**Fig. 1:**
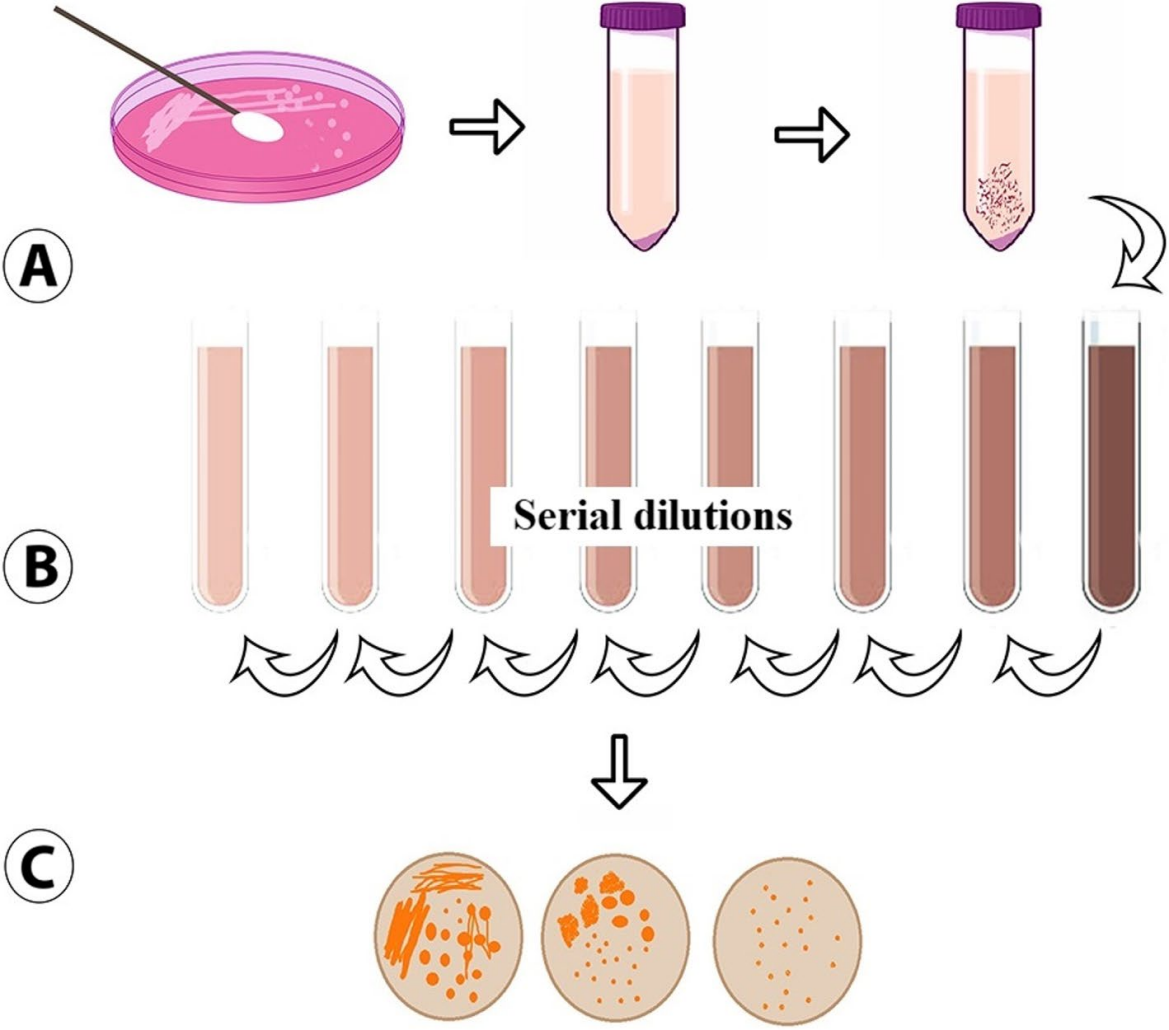
Schematic illustration of bacterial enumeration. *S. aureus* strains were cultured (**A**). Dilution series were schematized (**B**). The bacterial colonies in biofilm layers were observed (**C**)

Catheter pieces, which were rinsed three times with 0.9% NaCl for 30 min by shaking, were placed in control broth (Mueller–Hinton Broth, bioMérieux, Marcy l’Etoile, France) in different tubes which contain DAPT (2 mg/ml) (50 mg/lt Ca^+ 2^ added at physiological concentration), VAN (10 mg/ml), LIN (2 mg/ml), and combination with NAC (6 mg/ml), respectively for 24 h at 37 °C [18]. All antibiotic solutions, daptomycin (Novartis Health, Food and Agriculture Products Industry and Trade Inc., Istanbul, Turkey), vancomycin (Meditera Group, Izmir, Turkey), linezolid (Pfizer Ltd. Şti., Istanbul, Turkey), and NAC (Merc, Millipore, Germany), were prepared fresh from commercially available forms in accordance with the manufacturer’s recommendations. Catheter parts were washed with 0.9% NaCl, placed in 5 ml of 0.9% NaCl prepared in a different tube, and vortexed for 30 s, and then 10 μl of liquid was taken and inoculated on 5% sheep blood agar. Single dropped colonies were counted following 24 h of incubation at 37 °C [18]. All microbial steps were performed according to our previous study, and the method described in Fig. 1 is adapted from our previous work (Fig. 1) [19, 20].

### Electron microscopic analysis

Electron microscopic evaluation of biofilm on the material surface was performed using a scanning electron microscope (SEM) (JSM-7001F STEM, JEOL Ltd., Tokyo, Japan). Following the dried and coated with gold–palladium of the pieces, eight selected areas were investigated at × 10.000 magnification. The histological scoring system was used as in our previous work [20].

### Statistical analysis

The statistical analysis data was evaluated using the SPSS Ver. 21 software (IBM Corp., Armonk, NY). Data were expressed as mean ± SD. The significant differences among groups were assessed using a non-parametric test, the Kruskal–Wallis. Differences were considered statistically significant at *p* < 0.05.

## Results

### SEM visualization

**Fig. 2:**
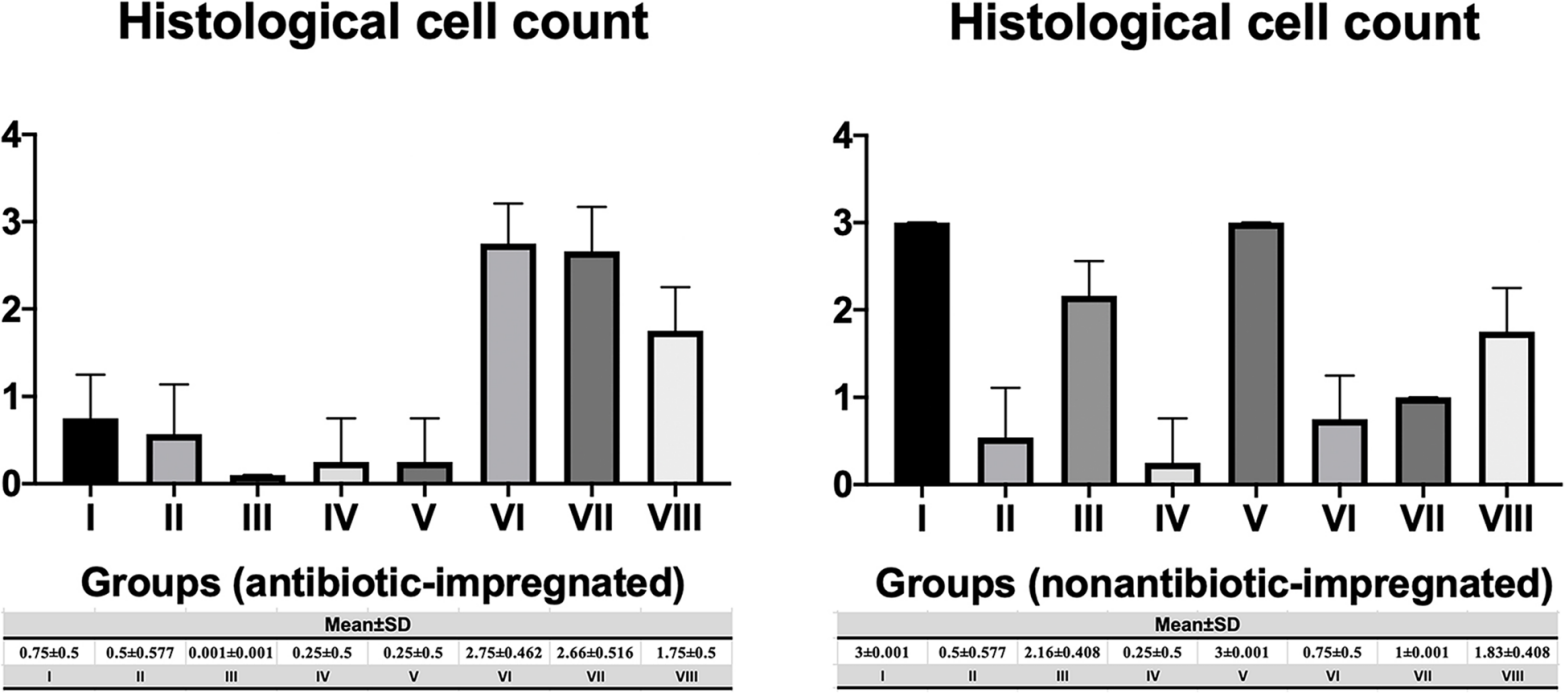
The graph shows the histological cell count of the antibiotic-impregnated and nonantibiotic-impregnated groups obtained from SEM analyses

**Fig. 3:**
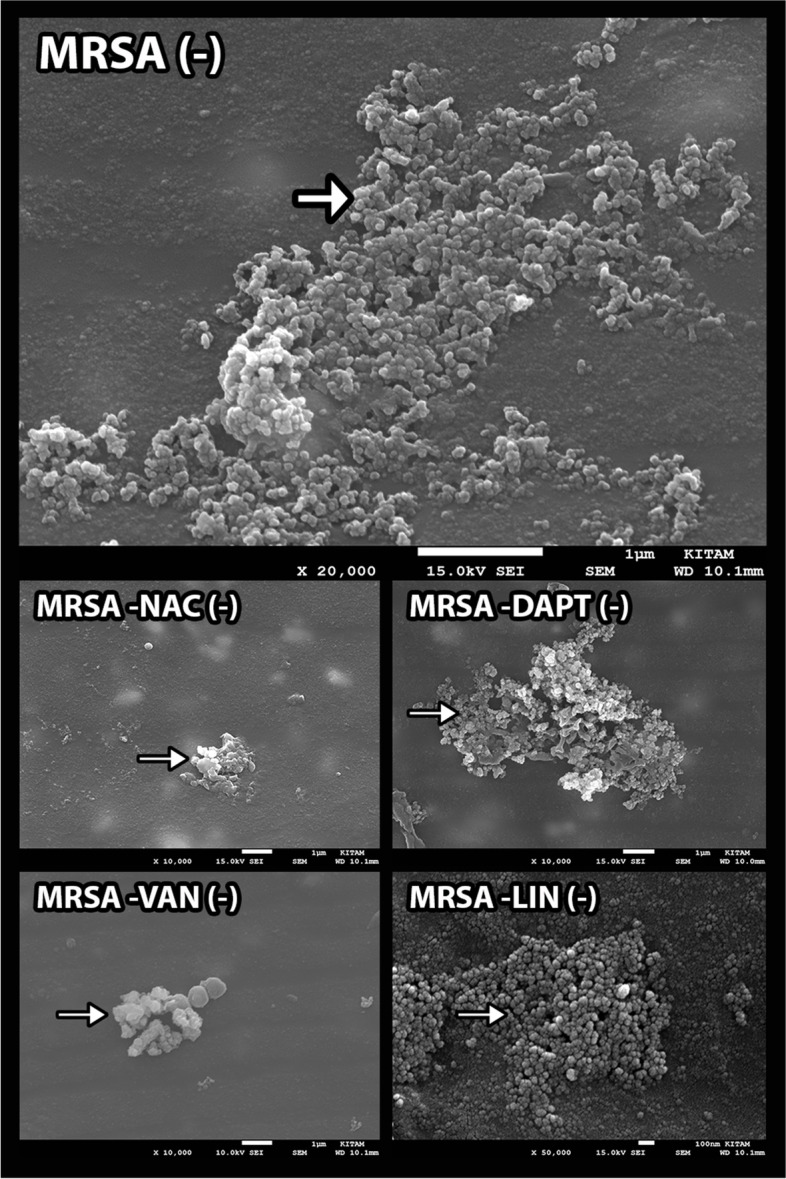
SEM images show the in vitro activity of relevant substances against *S. aureus* biofilms in nonantibiotic-impregnated (MRSA( −), MRSA-NAC ( −), MRSA-DAPT ( −), MSA-VAN ( −), and MRSA-LIN ( −)) catheters. Arrows indicate *S. aureus* colonies (original magnification: × 10.000)

**Fig. 4:**
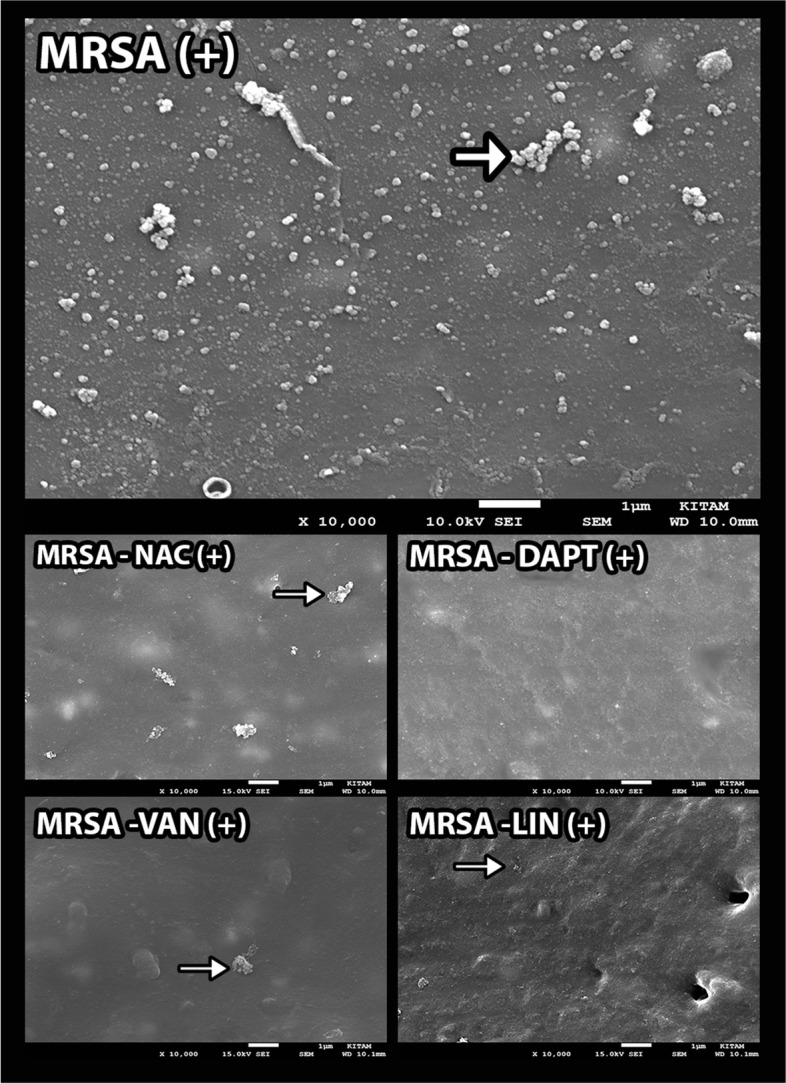
SEM images show the in vitro activity of relevant substances against *S. aureus* biofilms in antibiotic-impregnated (MRSA( +), MRSA-NAC ( +), MRSA-DAPT ( +), MRSA-VAN ( +), and MRSA-LIN ( +)) catheters. Arrows indicate *S. aureus* colonies (original magnification: × 10.000)

**Fig. 5:**
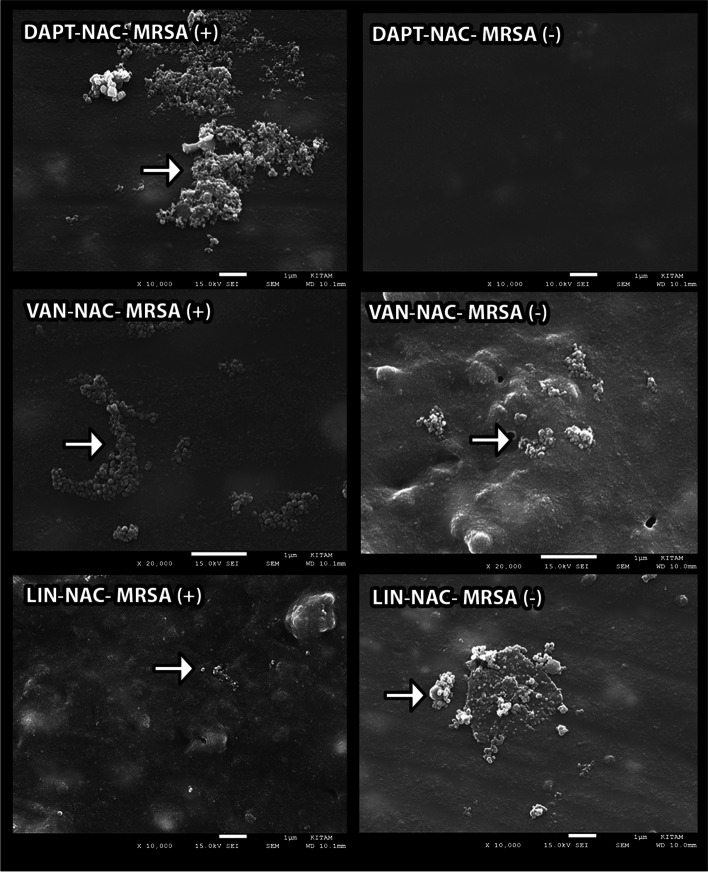
SEM images show the in vitro activity of relevant substances against *S. aureus* biofilms in both antibiotic-impregnated and nonantibiotic-impregnated ((DAPT- NAC- MRSA( +) and ( −), VAN-NAC-MRSA ( +), and ( −), LIN-NAC-MRSA ( +) and ( −)) catheters. Arrows indicate *S. aureus* colonies (original magnification: × 10.000)

Regarding the SEM evaluation, the colony numbers of antibiotic-impregnated catheters in groups VI, VII, and VIII were found higher than the other groups and statistically significant (*p* < 0.01), respectively. In addition, the number of colonies in nonantibiotic-impregnated catheters of groups I, III, V, and VIII was also found to be significantly higher (*p* < 0.01) compared to other groups, respectively. Furthermore, significant decreases in the number of colonies of the antibiotic-impregnated groups were observed compared to nonantibiotic-impregnated groups in terms of groups I, III, and V (*p* < 0.01), respectively. Contrarily, there were significant increases in the colony count of antibiotic-impregnated groups compared to nonantibiotic-impregnated groups in points of group VI and group VII (*p* < 0.01). However, there were no significant differences between the antibiotic-impregnated and nonantibiotic-impregnated samples in groups II, IV, and VIII (*p* > 0.05) (Figs. 2, 3, 4, and 5).

### Microbial colony counts

**Fig. 6:**
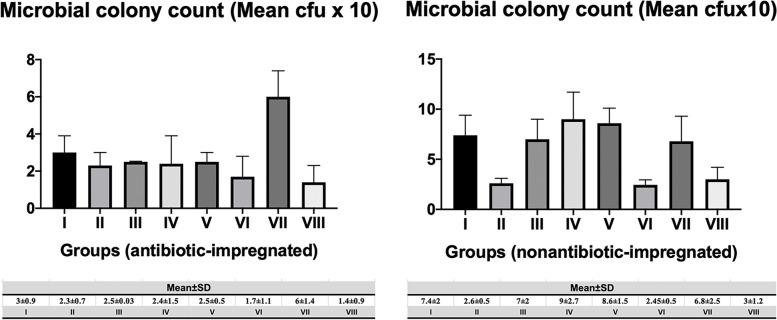
The graph shows the microbial colony count (mean cfu × 10) of the antibiotic-impregnated and nonantibiotic-impregnated groups

Regarding the microbial counts of the samples, there was a significant increase in the antibiotic-impregnated group VII compared to the other antibiotic-impregnated groups (*p* < 0.01). Additionally, there were also significant increases in the nonantibiotic-impregnated groups I, III, IV, V, and VII compared to the nonantibiotic-impregnated groups II, VI, and VIII (*p* < 0.01), respectively. In addition, there were significant differences between the antibiotic-impregnated and nonantibiotic-impregnated groups III, IV, and V (*p* < 0.01), respectively (Fig. 6).

## Discussion

A shunt is administered to control intracranial pressure. However, secondary infections can develop during ventricular drainage at about the rate of 15%. Currently, the treatment of shunt infections takes several weeks, depending on shunt removal and intravenous antibiotics [21]. Despite improvements in catheter technology, it is still a major concern for the patient and surgeon due to its complications [22]. Complications related to shunt surgery are clinically highly confronted with the prevalence ranging from 2 to 27%. In particular, they are the most common infections due to obstruction. Shunt-related infections can occur during or immediately after the surgical procedures [16, 23]. *Staphylococcus epidermidis* is the most common cause of shunt infections, followed by *S. aureus*. Enterococcus-related shunt infections are less common [2]. Empirical antimicrobial therapy of CSF shunt infections should include agents with bactericidal activity against most common pathogens that penetrate and colonize the central nervous system. For perioperative prophylaxis, anti-staphylococcal penicillin is replaced by VAN (10 mg/ml) due to rising MRSA rates.

According to our study, the number of colonies on the surface of nonantibiotic-impregnated shunts in MRSA (group I), MRSA + DAPT (group III), and MRSA + LIN (group V) groups significantly increased compared to antibiotic-impregnated groups (*p* < 0.01) in terms of histological cell count (Fig. 2). These findings were compatible with each other in terms of histological and microbiological colony counts. Regarding the microbial colony counts of antibiotic-impregnated and nonantibiotic-impregnated groups, the colony counts in the nonantibiotic-impregnated groups of DAPT (group III), VAN (group IV), and LIN (group V) were higher than the antibiotic-impregnated groups. These results suggested that rifampicin- and clindamycin-coated catheters, which have strong antistaphylococcal and antibiofilm activities, could be more effective in preventing infection than other substances used in the study. Antibiotic-impregnated systems have recently gained importance in reducing and preventing shunt infections. They showed activity against coagulase-negative *Staphylococcus*, *S. aureus* strains with decreasing gram-positive bacteria colonization [24, 25]. Although it has been shown that the antimicrobial activity in these catheters continues on the 127th day after the application, it has been reported that the risk of infection is reduced by 2.4 times and that the causative agent is not *Staphylococcus* type bacteria in patients who develop shunt infection in which catheters are used [26–28]. Some studies showed that it does not inhibit bacterial adhesion but reduces adherent bacteria by 100% within 48–52 h. In antibiotic-impregnated systems, 50% of the infection incidence was demonstrated by clinical studies [26, 29]. Additionally, while the infection in nonantibiotic-impregnated systems was 11.2% in a study, this ratio was 3.2% in patients who have antibiotic-impregnated catheter treatment [30]. Antibiotic-impregnated systems decrease shunt infections by preventing colonization, also observed in high-risk patients. In a retrospective study involving adult patients, the proportion of the infected patients in the nonantibiotic-impregnated group was 58%, while it was 50% in the antibiotic-impregnated group. However, there are few studies in the literature reporting no significant difference although antibiotic-impregnated catheters reduce the infection rate [31, 32].

MRSA meningitis is associated with a high mortality rate making the treatment difficult in patients with VAN allergy [33]. According to our results, VAN efficacy in nonantibiotic-impregnated shunts is similar to the antibiotic-impregnated shunts. Thus, our results confirmed that VAN alone is a potent treatment against MRSA infection. In addition, high concentrations of VAN by the intraventricular administration showed a long-lasting effect on staphylococcal biofilms [8]. However, as VAN is a large molecule compared to DAPT, its distribution may be insufficient and its low antibiofilm activity may cause a decrease in its potency in intraventricular administration in patients whose shunt cannot be removed. In our study, the high number of colonies in microbial examination could be explained by this while the histological colony count was low. In addition, although DAPT has a rapid bactericidal effect compared to VAN, it causes less inflammation since it exerts this effect without lysis [34]. However, there is a great need for clinical studies. Also, evaluating the viability of microorganisms using advanced techniques will make our data more meaningful.

On the contrary, the number of colonies in the NAC + VAN group (group VII) in antibiotic-impregnated catheters was found to be higher in the histological approach. A similar situation was also detected in the NAC + DAPT group (group VI). These results might be due to the antagonist effect of NAC with both rifampicin and clindamycin in the catheter. However, synergy or antagonism could not be examined by applying a microbiological method that is the limitation of our study. Although additive and synergistic effects of NAC with VAN on staphylococcal bacteria have been demonstrated, no study has been found in the literature concerning its interaction with rifampicin and clindamycin. Low colony count in nonantibiotic-impregnated NAC + DAPT (group VI) and NAC + VAN (group VII) groups suggested that it may be due to the synergistic or additive activity of NAC with DAPT and VAN. According to our results, it may be concluded that NAC or DAPT can be used in patients with VAN allergy, although systemic transmission of DAPT to the CNS is not good, it might be administered intraventricularly or intrathecally. Additionally, a combination of NAC and DAPT can be considered more effective in the use of nonantibiotic-impregnated catheters that are not coated with rifampicin and clindamycin. However, a systematic evaluation of the clinical efficacy of this combination is required.

Although the primary means of preventing CSF shunt infections is to remove the shunts, the patient’s tolerance to the surgical operation may not make this possible. In this regard, the main antibiotic was thought to be in non-surgical treatment. In particular, LIN could abolish MRSA biofilms by intravenous or oral administration. Brown et al. reported that LIN and VAN might be effective without shunt removal against staphylococcal shunt infections [5]. LIN can achieve therapeutic CSF levels by intravenous administration compared to VAN, though the high colony count detected in histological and microbial examination in the presence of NAC alone or in combination with LIN in the nonantibiotic-impregnated catheters in our study can be explained by the bacteriostatic effect of LIN on MRSA and the instability of this light-sensitive molecule in the in vitro environment.

In conclusion, in order to prevent shunt infections with MRSA, which has a high morbidity and mortality, our study may recommend the use of rifampicin- and clindamycin-coated catheters or the use of intraventricular or intrathecal NAC and DAPT combination for the prevention and treatment of infections that may occur if the nonantibiotic-impregnated catheters used in the patient whose shunt cannot be removed. A greater number of controlled trials comparing different treatments for shunt infections are needed.

## Data Availability

Not applicable.

## Abbreviations

MRSA: Methicillin-resistant *Staphylococcus **aureus*
DAPT: Daptomycin
VAN: Vancomycin
LIN: Linezolid
NAC: N-Acetylcysteine
SEM: Scanning electron microscope
*S. **aureus*: *Staphylococcus* *aureus*
CSF: Cerebrospinal fluid
